# Real-time occupational radiation dosimetry in anesthesiology personnel during fluoroscopy-guided interventions: a single-center descriptive and exploratory study

**DOI:** 10.3389/fpubh.2026.1924968

**Published:** 2026-10-28

**Authors:** T. M. Axakali, M. A. Kouri, P. Georgakis, E. Bournaki, V. Tanou, E. Kounadi, C. Michail, N. Kalyvas, I. Valais, G. Fountos

**Affiliations:** 1 Radiation Physics Materials Technology and Biomedical Imaging Laboratory (ΑΚΤΥΒΑ), Department of Biomedical Engineering, University of West Attica, Egaleo, Athens, Greece; 2 Medical Physics, General Hospital GHA Korgialeneio Mpenakeio-Hellenic Red Cross, Athens, Greece; 3 Medical Physics Unit, 2nd Department of Radiology, Medical School, National and Kapodistrian University of Athens, Athens, Greece; 4 Αnesthesiologists’ Department, General Hospital GHA Korgialeneio Mpenakeio-Hellenic Red Cross, Athens, Greece

**Keywords:** anaesthesiologists, electronic personal dosimeters, fluoroscopy-guided interventions, occupational health, occupational radiation exposure, radiation protection, real-time dosimetry

## Abstract

Anesthesia personnel participating in fluoroscopy-guided interventions are routinely exposed to ionizing radiation; however, their occupational radiation burden remains insufficiently characterized. This single-center descriptive and exploratory study investigated occupational ionizing radiation exposure among anesthesiology personnel using real-time personal dosimetry. A total of 134 procedures across multiple interventional specialties were evaluated using the HERADO radiation management platform and ALMAR active personal dosimeters. Mean over-apron personal dose equivalent Hp(10) ranged from 0.010 ± 0.009 mSv to 0.025 ± 0.011 mSv per procedure. Descriptive differences in occupational exposure were observed across procedural categories; however, these differences should not be attributed independently to procedural category because relevant technical, geometric, shielding, and workflow factors were not fully quantified. The findings demonstrate the value of real-time personal dosimetry for procedure-level characterization of occupational exposure and may inform radiation-protection strategies in fluoroscopy-guided clinical practice.

## 1 Introduction

Ionizing radiation is classified by the International Agency for Research on Cancer (IARC) as a Group 1 carcinogen (1). Occupational exposure to ionizing radiation has been associated with stochastic biological effects, including radiation-induced malignancies (33), and tissue reactions in radiosensitive organs such as the eye lens (2). To limit these risks, the International Commission on Radiological Protection (ICRP) has established occupational dose limits and recommends the optimization of radiation protection through the application of the As Low As Reasonably Achievable (ALARA) principle (3–5, 36).

In fluoroscopy-guided procedures, healthcare professionals are exposed mainly to scatter radiation that originates from patients (6). Although the advances in fluoroscopic technology and modern radiation protection practices have reduced occupational exposure, radiation remains an important consideration for the personnel working in ionizing radiation fields (7, 34).

The majority of studies evaluating occupational exposure during interventional procedures have focused primarily on the main procedural operators, while exposure among anesthesiology personnel has been extensively characterized (7–9). Anesthesiology personnel represent a distinct occupational group because their role, positioning within the procedure room, proximity to the radiation source, and duration of presence may differ from those of the primary operator. Conventional occupational monitoring commonly relies on passive dosimeters that provide cumulative dose measurements over defined monitoring periods but offer limited information regarding procedure-specific exposure patterns. Consequently, evidence characterizing occupational exposure among anesthesiology personnel across different fluoroscopy-guided procedural categories using real-time dosimetry remains limited (8).

The primary objective of the present study was to characterize procedure-specific occupational radiation exposure among anesthesiology personnel participating in fluoroscopy-guided interventions using individualized real-time dosimetry, with occupational dose measured at chest level over the lead apron as the primary outcome. The secondary objectives were to descriptively characterize the occupational dose in relation to fluoroscopy time, dose area product (DAP), and body mass index (BMI), and to compare occupational doses across different procedural categories. The study addressed whether occupational radiation exposure among anesthesiology personnel varies across fluoroscopy-guided procedural categories and is associated with procedural radiation parameters.

## 2 Materials and methods

### 2.1 Study design and clinical procedures

This single-center prospective observational study was conducted at the General Hospital Korgialeneio-Benakeio Hellenic Red Cross, Athens, Greece, which is a tertiary-care institution that conducts a broad spectrum of fluoroscopy-guided diagnostic and therapeutic interventions. Data were collected prospectively between January and November 2025 during routine clinical practice. This study evaluated occupational radiation exposure among anesthesiology personnel performing routine fluoroscopy-guided procedures under clinical conditions.

The Scientific and Ethics Committee (Approval No. [3697]) and the Board of Directors (Approval No. [5,182/17.02.2025]) of the General Hospital Korgialeneio-Benakeio Hellenic Red Cross approved the study protocol. The Head of the Department of Anaesthesiology granted permission to conduct the study and monitor occupational radiation exposure. As the study was purely observational, involved no modification of routine clinical practice, and collected occupational dosimetry data without affecting patient care, the Ethics Committee waived the requirement for individual informed consent from anesthesiology personnel. Occupational radiation exposure data were collected during routine clinical practice across multiple clinical procedures including neurointerventional, endovascular, interventional cardiology, endoscopic retrograde cholangiopancreatography (ERCP), urological, and orthopedic interventions. No modifications were made to procedural workflow, imaging protocols, or radiation protection practices for the purposes of the study.

Individualized real-time dosimetry was introduced using the HERADO active electronic personal dosimeters (9). The radiation exposure of the anesthesiologists was recorded for each procedure and analyzed alongside procedural and technical exposure parameters.

### 2.2 Study population

The study population consisted of anesthesiologists’ personnel who are daily exposed to radiation due to the fluoroscopy-guided procedures that they participate in during routine clinical practice. The study focused on anesthesiologists’ personnel, because their role in interventional procedures differs from that of the primary operator, resulting in different positioning within the procedure room and potentially different exposure patterns (10).

Five senior anesthesiologists participated in occupational monitoring during the study period, and each anesthesiologists contributed to multiple fluoroscopy-guided procedures. The analytical dataset was structured at the procedural level. Although the total number of procedures was recorded for each procedural category, individual personnel identifiers linking each procedure to specific anesthesiologists were not retained in the analytical dataset. Consequently, the number of procedures contributed by each anesthesiologist within each procedural category cannot be retrospectively determined. Repeated observations from the same participant could therefore not be identified or modeled at the individual level.

The participants were included when:

- were active in the fluoroscopy-guided procedures,
- were continuously monitored with HERADO real-time dosimeters, and
- complete procedural and dosimetric data were collected, including fluoroscopy time, dose area product (DAP), and relevant imaging parameters.

Procedures were excluded when dosimetric monitoring was interrupted or when key exposure data were missing.

Data collection was conducted during routine clinical activity without modifying medical practice. Consequently, this study reflects occupational exposure under the continuous daily routine, including variations in procedure type, workflow, staff positioning, and the different use of all the radiation protection measures.

### 2.3 Procedural characteristics

The difference between fluoroscopy-guided procedures was determined by the clinical speciality and the procedural type. For each intervention, procedural and technical metadata were systematically recorded to support multidimensional exposure characterization. Collected variables included fluoroscopy time, DAP, tube voltage (kVp), tube current-time (mAs), patient body mass index (BMI), and anesthesiologists’ procedural positioning where available.

These parameters were selected because they represent established determinants of occupational radiation burden and allow interpretation of occupational exposure patterns (11).

A total of 134 fluoroscopy-guided procedures were included, distributed across neurointerventional (*n* = 32), interventional cardiology (*n* = 10), endovascular (*n* = 24), ERCP (*n* = 23), urological (*n* = 34), and orthopedics (*n* = 11) categories.

### 2.4 Occupational radiation monitoring

The HERADO monitoring system was implemented to provide real-time monitoring of radiation exposure during fluoroscopy-guided interventional procedures and to directly attribute recorded occupational dose to specific procedural events.

The ALMAR is a battery-powered active personal dosimeter designed for continuous radiation monitoring. Depending on the incorporated sensor configuration, ALMAR can measure different radiation components, including X-ray/gamma radiation, neutrons, electrons, and heavy ions. For the X-ray/gamma configuration used in the present study, the manufacturer reports a dose measurement range of 0.65 μSv—10 Sv and a dose-rate measurement range of 1 μSv/h—10 Sv/h. The device weights 25 g and has a battery life of approximately 14 days. The device stores recorded measurements in its memory and transfers them to the HERADO platform via Wi-Fi. According to the manufacturer’s specifications, the dosimeter complies with EN 61526 requirements. Unlike conventional passive dosimeters, which provide cumulative dose measurements over extended monitoring periods, active dosimetry enables real-time monitoring and procedure-specific characterization of dynamic occupational exposure patterns (9).

The HERADO platform assigns each sensor to a specific user, allowing recorded measurements to be associated with the corresponding wearer and retrieved for defined monitoring periods. According to the manufacturer’s technical documentation, the X-ray/gamma configuration of ALMAR used in the present study uses a silicon-based detector and measures the personal dose equivalent Hp(10). The manufacturer reports an energy-response range of 3 keV-100 MeV, angular dependence of approximately 5%, and high stability in pulsed radiation fields. The manufacturer also reports dose accuracy of ± 5% and dose-rate linearity within 5% under Cs-137 reference irradiation, as well as calibration/testing performed at Los Alamos and HIMAC. The manufacturer-issued Declaration of Conformity lists EN 61526:2013 as an applicable radiation-protection standard. These performance characteristics are manufacturer-reported specifications and were not independently determined by the investigators.

For each procedure, the start and end times were recorded, and the corresponding dosimetric measurements were retrieved from the HERADO platform for the defined procedural time interval, enabling procedure-specific attribution of the recorded occupational dose. Zero readings were retained as recorded and were not treated as missing data. A zero reading indicates that no dose above the manufacturer-reported lower detectable level of approximately 0.65 μSv was recorded during the corresponding monitoring interval and should not be interpreted as evidence of an absolute absence of radiation exposure (9).

The anesthesiology personnel participating in the interventions were equipped with the ALMAR dosimeters worn externally over the lead apron at chest level throughout the monitored procedures. A standardized external position was used to ensure consistent dosimeter placement across procedures. The general principle of over-apron monitoring is consistent with established occupational radiation-protection practice for personnel working in fluoroscopy-guided interventions (35). In the present study, the recorded Hp(10) values represent personal dose equivalent measured at the external chest-level dosimeter position and were used to characterize occupational exposure to scattered radiation. Because only a single dosimeter was worn externally over the protective apron, the recorded Hp(10) values were not interpreted as direct estimates of whole-body effective or organ absorbed dose (37).

No independent validation of the individual dosimeters used in this study against a calibrated reference dosimeter under diagnostic X-ray beam qualities representative of the clinical pulsed-fluoroscopy conditions was performed, and no study-specific measurement-uncertainty assessment was conducted.

Radiation exposure measurements were continuously recorded and automatically stored within the HERADO platform. For each monitored procedure, measurements corresponding to the predefined procedural time interval were subsequently retrieved and used for procedure-level characterization of occupational exposure (12).

### 2.5 Exposure parameters

To characterize procedural, technical, and patient-related factors relevant to occupational radiation exposure, available exposure parameters were recorded for each fluoroscopy-guided intervention based on procedural documentation.

DAP and fluoroscopy time were recorded from the fluoroscopy system (C-arm) records. Technical imaging parameters, including kVp and mAs, and patient BMI were recorded when available, given their potential influence on occupational radiation exposure (13–15). In addition, the positioning of anesthesiology personnel relative to the radiation source and patient was documented descriptively whenever available; however, positioning data were not systematically available for quantitative analyses (16).

Detailed procedure-level information regarding the use of ceiling-suspended shields and table-side curtains, anesthesiologist distance and position relative to the radiation source, beam geometry and angulation, imaging mode, shielding configuration, and workflow-related factors was not systematically recorded and was therefore unavailable for quantitative analysis. Missing supplementary variables were not imputed. Consequently, these factors could not be incorporated into an adjusted analysis, and the observed exposure patterns were interpreted descriptively without attributing them to an independent effect of procedural category.

### 2.6 Statistical analysis

Statistical analysis was performed using Microsoft Excel (Microsoft Office 2021). Procedural characteristics and occupational exposure were summarized across the different fluoroscopy-guided intervention categories. Continuous variables were reported as mean ± standard deviation (SD), while for each procedural group, the median, minimum, and maximum values were calculated to characterize the variability that exists in occupational exposure.

Given the repeated participation of individual anesthesiologists across multiple procedures and the absence of participant identifiers in the analytical dataset, within-participant dependence could not be identified or explicitly modeled. Therefore, participant-adjusted inferential analysis was not possible, and the statistical analysis was restricted to descriptive characterization of the procedure-level data. No inferential statistical significance testing or associated *p*-values were reported.

Occupational radiation dose measured using the HERADO system was considered the primary outcome variable. Occupational dose, fluoroscopy time, DAP, and BMI were summarized descriptively across the six procedural categories. Graphical distributions were used to illustrate procedure-level variability and exposure patterns across the investigated procedural categories.

Zero values recorded by the dosimetry system were retained in the descriptive analysis and were not treated as missing data. These values indicated that no measurable occupational dose was recorded by the dosimeter during the corresponding procedure and should not be interpreted as evidence of an absolute absence of radiation exposure.

Each observation represented a single fluoroscopy-guided procedure. Since participant identifiers were not retained in the analytical dataset, repeated observations from the same anesthesiologist could not be identified, and participant-level or repeated-measures inference could not be performed.

## 3 Results

### 3.1 Characteristics of the study dataset

A total of 134 fluoroscopy-guided procedures met the inclusion criteria and were included in the final analysis. The study included six procedural categories representing different interventional specialties with distinct imaging requirements and radiation exposure profiles.

Interventional cardiology procedures had the highest mean fluoroscopy time (34.04 min), followed by neurointerventional (20.05 min) and endovascular procedures (15.94 min). In contrast, orthopedic, Urological, and ERCP procedures showed shorter fluoroscopy time, with mean values of 1.57 min, 2.52 min, and 3.33 min, respectively (Figure 1).

**Figure 1:**
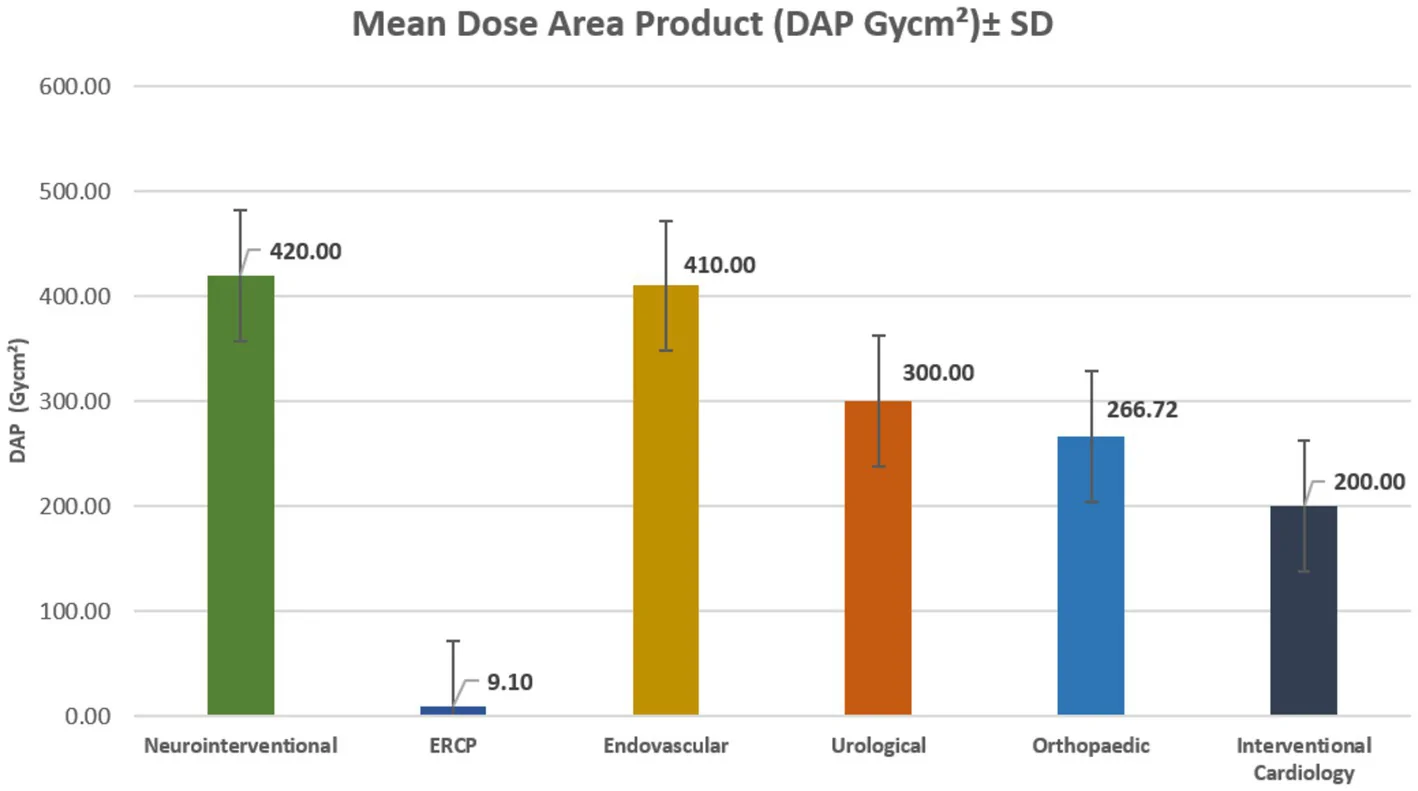
Mean dose area product (DAP) across the investigated fluoroscopy-guided procedural categories. Numbers above the bars indicate mean DAP values, and error bars represent standard deviation (SD). Sample sizes were: neurointerventional, *n* = 32; ERCP, *n* = 23; endovascular, *n* = 24; urological, *n* = 34; orthopedic, *n* = 11; and interventional cardiology, *n* = 10.

Mean patient BMI was similar across all procedural categories, ranging from 26.4 **±** 8.1 kg/m^2^ in urology to 28.4 ± 4.0 kg/m^2^ in interventional cardiology (Figure 2).

**Figure 2:**
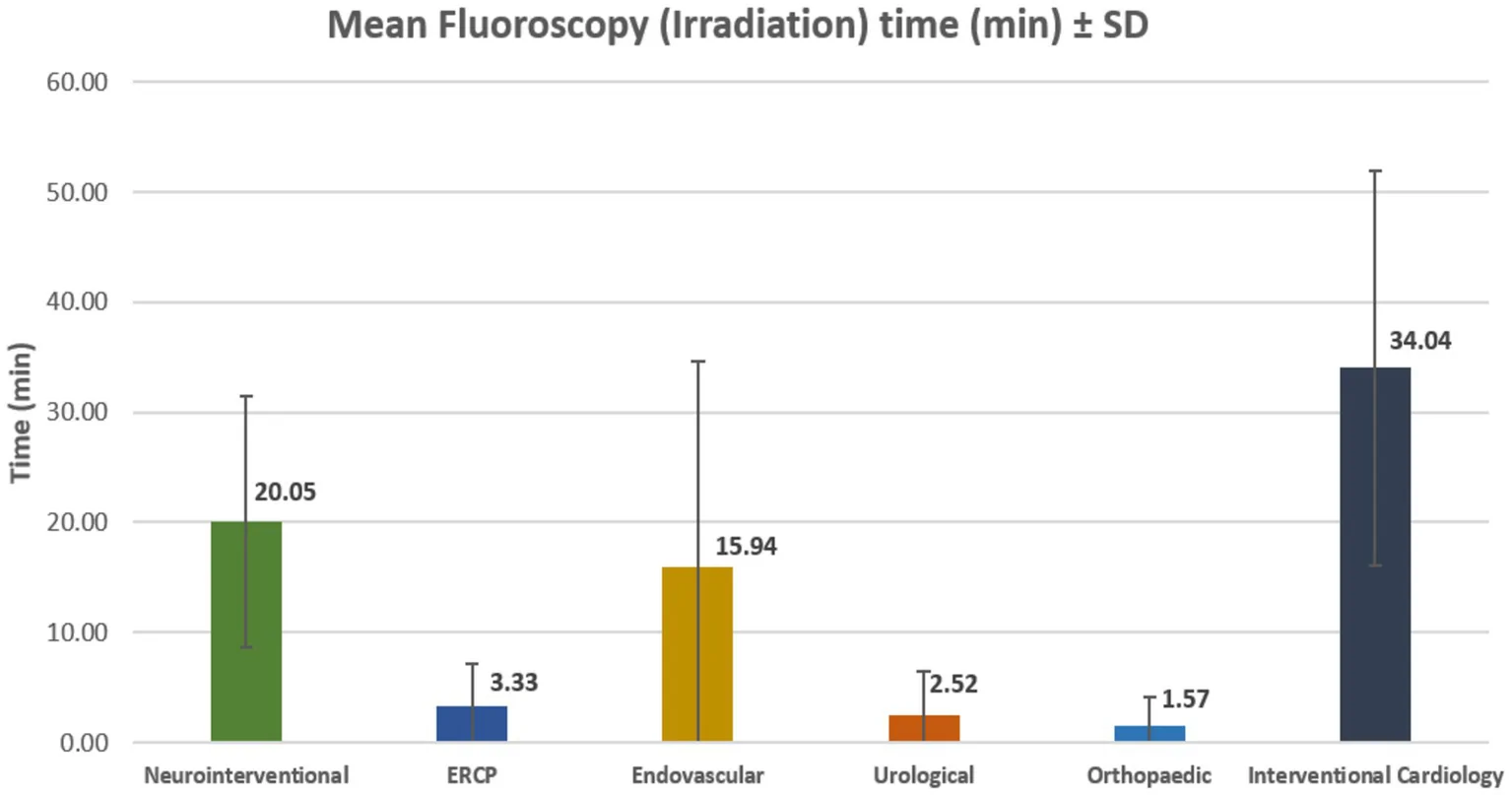
Mean fluoroscopy time across the investigated fluoroscopy-guided procedural categories. Numbers above the bars indicate mean fluoroscopy time (minutes), and error bars represent standard deviation (SD). Sample sizes were as follows: neurointerventional, *n* = 32; ERCP, *n* = 23; endovascular, *n* = 24; urological, *n* = 34; orthopedic, *n* = 11; and interventional cardiology, *n* = 10.

Mean DAP values varied across six procedural categories. Neurointerventional and endovascular procedures showed the highest mean DAP values, whereas ERCP procedures showed the lowest values. Table 1 presents the detailed characteristics for all the above.

**Table 1:** Mean BMI, fluoroscopy time, and DAP per interventional procedure included in the final datasheet.

Procedure category	N	BMI (Mean ± SD) (kg/m^2^)	Fluoroscopy time (min)	DAP (Gy·cm^2^)
Neurointerventional	32	26.4 ± 4.3	20.05	420
ERCP	23	27.3 ± 9.6	3.33	9.1
Endovascular	24	27.9 ± 6.3	15.94	410
Urological	34	26.3 ± 8.1	2.52	300
Orthopedic	11	26.5 ± 3.1	1.57	266.72
Interventional cardiology	10	28.4 ± 4.0	34.04	200

### 3.2 Occupational radiation exposure

Occupational radiation exposure differed across procedural categories. The mean occupational doses recorded using the HERADO real-time dosimetry system ranged from 0.010 **±** 0.009 mSv to 0.025 ± 0.011 mSv per procedure.

The highest mean occupational dose was observed during interventional cardiology procedures (0.025 ± 0.011 mSv), followed by endovascular interventions (0.022 ± 0.012 mSv) and neurointerventional procedures (0.019 ± 0.013 mSv). In contrast, ERCP procedures demonstrated the lowest mean occupational exposure (0.010 ± 0.009 mSv). The mean occupational dose observed in interventional cardiology was 2.5-fold (150%) higher than the dose recorded in ERCP procedures.

Variability within each procedural category was observed, as reflected by the median and minimum-maximum values reported in Table 2. The highest occupational doses reached 0.036 mSv for interventional cardiology procedures, 0.034 mSv for endovascular interventions, and 0.020 mSv for ERCP procedures. Table 2 presents detailed occupational dose measurements, including mean ± SD, median, and minimum-maximum values for each procedural category (Figure 3).

**Table 2:** Procedure-specific occupational radiation exposure measured using the HERADO real-time dosimetry system.

Procedure category	HERADO dose (mSv) Mean ± SD	Median (mSv)	Minimum (mSv)	Maximum (mSv)
Neurointerventional	0.019 ± 0.013	0.016	0.007	0.032
ERCP	0.010 ± 0.009	0.007	0.001	0.020
Endovascular	0.022 ± 0.012	0.022	0.010	0.034
Urological	0.018 ± 0.013	0.015	0.005	0.031
Orthopedic	0.016 ± 0.010	0.010	0.006	0.027
Interventional cardiology	0.025 ± 0.011	0.027	0.014	0.036

**Figure 3:**
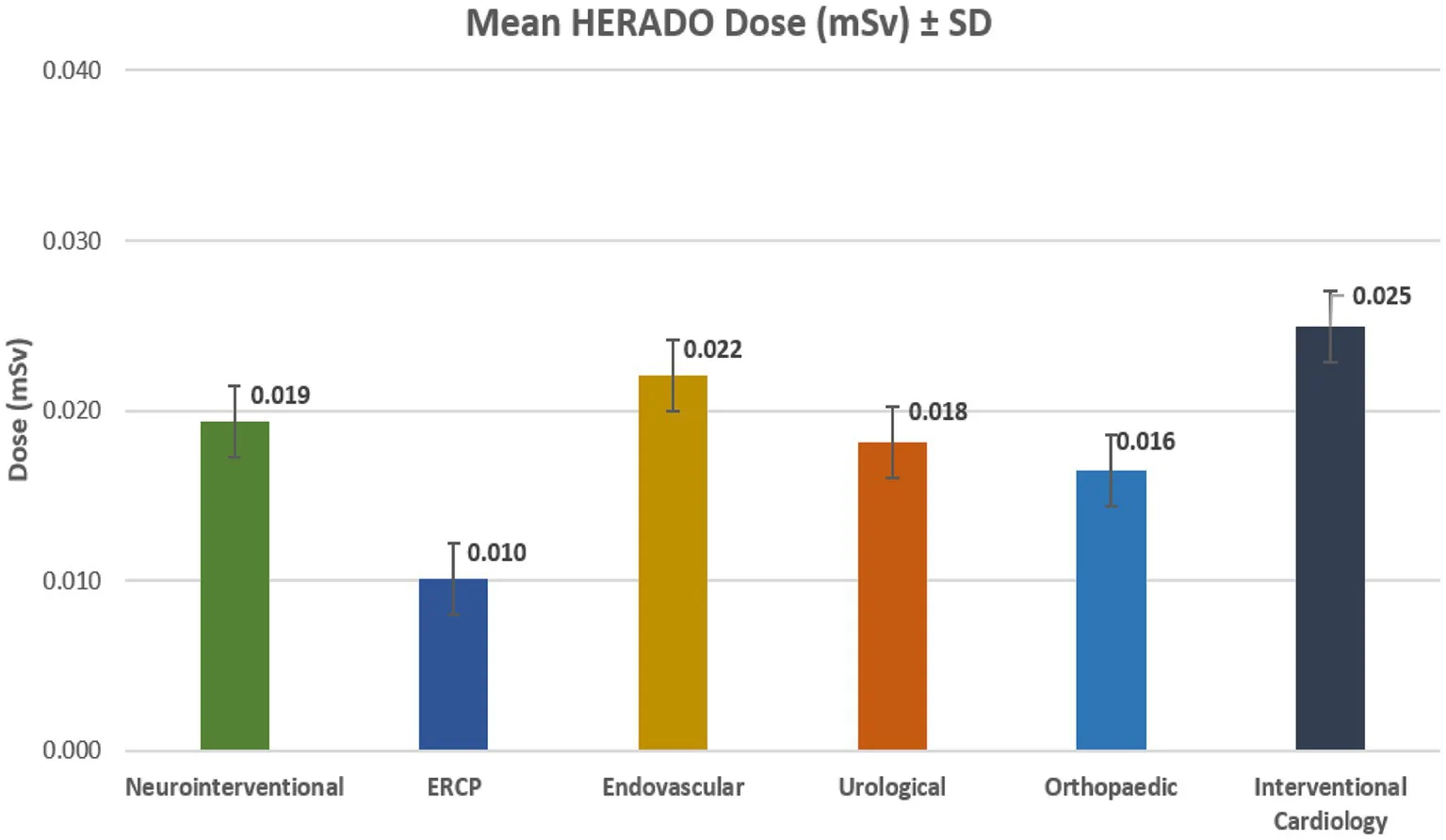
Mean over-apron personal dose equivalent, $H_{p}(10)$ , measured using the HERADO real-time dosimetry system across the investigated fluoroscopy-guided procedural categories. Numbers above the bars indicate the mean $H_{p}(10)$ values in mSv, and error bars represent standard deviation (SD). Sample sizes were as follows: neurointerventional, *n* = 32; ERCP, *n* = 23; endovascular, *n* = 24; urological, *n* = 34; orthopedic, *n* = 11; and interventional cardiology, *n* = 10.

## 4 Discussion

The main contribution of this single-center descriptive and exploratory study is the characterization of occupational radiation exposure among anesthesiology personnel, a professional group that has received less attention in the radiation-protection literature than primary interventional operators. By applying real-time personal dosimetry across six fluoroscopy-guided procedural categories, the study enabled descriptive characterization of occupational exposure patterns across multiple interventional specialties under routine clinical conditions. Extrapolation of cancer risk estimates from short-term radiation exposure scenarios requires careful consideration of the underlying assumptions and uncertainties (38).

The study recorded occupational radiation exposure among anesthesiology personnel across all investigated procedural categories (17). The mean recorded dose per procedure was substantially below the annual occupational exposure limits recommended by the ICRP; the observed procedure-level doses varied across the investigated clinical settings. These descriptive findings highlight the relevance of occupational radiation exposure among anesthesiology personnel, which is a comparatively understudied group in the radiation-protection literature (4, 18, 19). Unlike primary interventional operators, anesthesiology personnel have an indirect procedural role and may occupy different positions relative to the patient and radiation source while providing anesthetic monitoring and patient care, potentially resulting in distinct patterns of exposure to patient-scattered radiation.

The descriptive findings of the present study indicate that fluoroscopy time alone may not fully characterize occupational radiation exposure. Occupational dose, fluoroscopy time, and DAP showed variable procedure-level patterns across the procedural categories investigated. These observations suggest that occupational exposure may be influenced by multiple aspects of procedural radiation use and clinical workflow rather than fluoroscopy duration alone. This interpretation is consistent with previous studies indicating that occupational exposure may also vary according to procedural and technical conditions, including staff positioning, distance from the radiation source, beam angulation, and shielding practices (4, 7, 20).

Notably, the highest mean occupational dose in the present dataset was observed during interventional cardiology procedures, although this category did not show the highest mean DAP. This represents an unadjusted descriptive observation and should not be interpreted as evidence of an independent effect of procedural category. Occupational exposure may be influenced by several interrelated procedural and technical factors, including equipment characteristics, imaging parameters, beam geometry and angulation, procedure duration, staff positioning and distance from the radiation source, shielding configuration, and clinical workflow. As these factors were not systematically quantified and could not be incorporated into an adjusted analysis, the present dataset cannot determine their respective contributions to the observed exposure pattern.

Staff positioning may be associated with occupational radiation exposure; however, positioning data were not systematically available or quantitatively analyzed in the present study. Figure 4 provides a conceptual schematic of the predefined positions occupied by anesthesiology personnel relative to the patient and fluoroscopy system during clinical practice. Positions 1–3 represent the predefined working locations illustrated in the schematic, with positions 1 and 3 being the most frequently observed. As exact staff-to-patient and staff-to-X-ray-source distances were not systematically measured and no position-specific dosimetric analysis was performed, the relationship between staff positioning and procedure-level occupational exposure could not be quantitatively evaluated.

**Figure 4:**
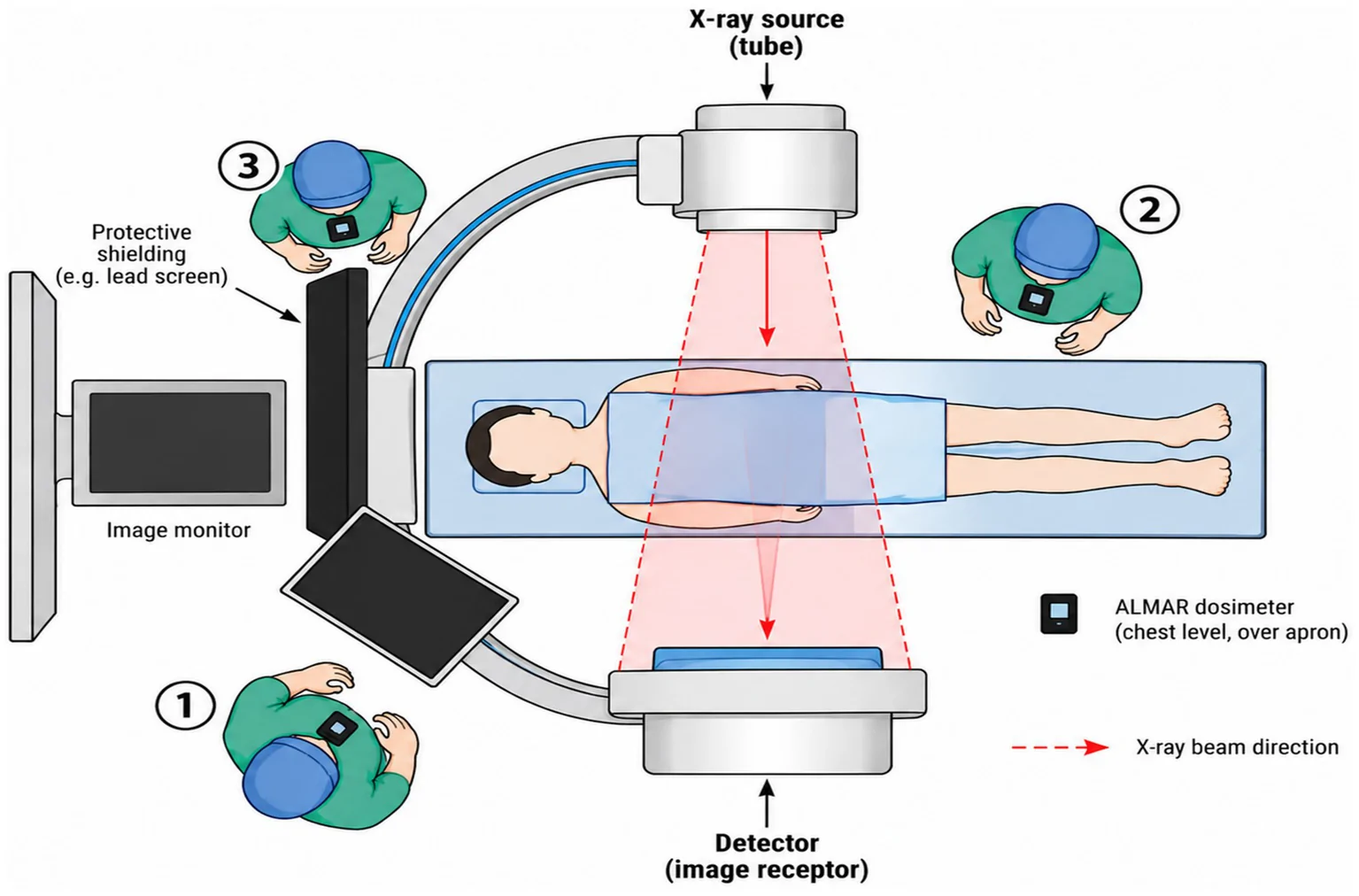
Conceptual top-down schematic of the predefined working positions of anesthesiology personnel relative to the patient and the C-arm fluoroscopy system. The X-ray source, detector, beam direction, patient, anesthesiology personnel, ALMAR dosimeter worn externally over the lead apron at chest level, and protective shielding are indicated. Positions 1–3 represent the predefined working locations observed during routine clinical practice, with positions 1 and 3 being the most frequently observed. Exact staff-to-patient and staff-to-X-ray-source distances were not systematically measured; therefore, distances and spatial relationships are schematic and not to scale. No spatial dose measurements were performed, and the illustration does not represent a quantitative dose map.

Conventional passive dosimetry remains essential for regulatory compliance and long-term occupational monitoring. Real-time electronic dosimetry provides additional temporal and procedure-specific information that complements conventional passive monitoring, which enables occupational exposure to be characterized at the level of individual interventions and facilitates the identification of procedure-specific exposure patterns (21, 22).

The integration of real-time dosimetry and procedure-specific exposure characterization may provide complementary information for occupational radiation assessment (23). These approaches may contribute to the development of radiation-protection strategies and improve occupational safety in fluoroscopy-guided procedures (24).

### 4.1 Findings in the context of current evidence

Occupational radiation exposure among anesthesiology personnel has been less extensively investigated than exposure among primary interventional operators (25, 26). Previous studies have demonstrated that anesthesiologists are exposed to scatter radiation originating primarily from the patient and that exposure may vary based on factors such as procedural characteristics, the positioning of professionals, and the radiation protection measures implemented. In the present study, occupational exposure also varied across procedural categories, with the highest mean dose observed in interventional cardiology (0.025 mSv per procedure), followed by endovascular (0.022 mSv) and neurointerventional procedures (0.019 mSv). This procedure-dependent pattern is broadly consistent with previous evidence identifying cardiac, endovascular, and neurointerventional environments as relevant sources of occupational exposure for anesthesiology personnel (27, 28). Previous studies have similarly reported measurable per-procedure occupational exposure among anesthesiologists in these interventional settings, although reported doses vary across studies. Direct comparison of absolute dose values between studies should be interpreted cautiously because of the differences in dosimeter position, procedural complexity, fluoroscopy equipment, staff positioning, shielding practices, and clinical workflow.

In addition to occupational dosimetry, structured radiation-protection education and training represent important components of occupational safety programs for healthcare professionals working in radiation environments (29, 30).

### 4.2 Future directions, strengths, and limitations

Future studies incorporating organ-specific dosimetry and long-term exposure monitoring may provide additional information regarding cumulative radiation exposure, particularly for radiosensitive organs such as the eye lens and thyroid. Such approaches should complement, rather than replace, conventional personal dosimetry by providing additional information on organ-specific dose distribution and cumulative exposure (31, 32).

Certain limitations should also be considered. First, these data were obtained from a single tertiary-care institution. Although the study included a broad spectrum of fluoroscopy-guided procedures, local practices, equipment, and radiation protection protocols may influence occupational radiation exposure. Consequently, the exposure patterns observed in the present study may not be directly generalizable to other institutions with different procedural practices, equipment, staffing arrangements, or radiation protection strategies.

A further limitation of the present study is the heterogeneity of the investigated procedural categories. Detailed procedure-level information on potential confounding factors, including equipment settings, beam geometry and angulation, staff position and distance from the radiation source, shielding configuration, and procedural workflow, was not systematically available. Although fluoroscopy time and DAP were recorded and additional parameters were documented when available, these potential confounding factors could not be incorporated into an adjusted analysis. Accordingly, the observed differences between procedural categories represent unadjusted descriptive differences within the present dataset and cannot be attributed independently to procedure type.

Furthermore, the number of procedures was not evenly distributed across categories. Some procedural groups had relatively small sample sizes, particularly interventional cardiology and orthopedic groups. These limited sample sizes reduce the precision and stability of category-specific descriptive estimates. Consequently, the descriptive ranking of procedural categories based on mean occupational dose estimates may be influenced by unequal sample sizes and within-category procedural variability and should therefore be interpreted with caution. In addition, the five participating anesthesiologists may have contributed measurements from multiple procedures and across procedural categories. The analytical dataset did not retain individual participant identifiers linking each procedure to a specific anesthesiologist. Consequently, repeated observations from the same participant could not be identified, the number of procedures contributed by each anesthesiologist within each procedural category could not be retrospectively determined, and within-participant dependence could not be modeled. Participant-adjusted inferential analysis was therefore not possible, and the present results were restricted to descriptive procedure-level characterization.

Occupational exposure was measured using electronic real-time dosimeters positioned externally over the protective lead apron at the chest level, providing the personal dose equivalent Hp(10) at the monitoring position. These measurements should not be interpreted as direct measurements of the absorbed dose to specific organs.

A further consideration concerns the metrological characterization of the active personal dosimeters. Although ALMAR is a commercially manufactured active personal dosimeter with manufacturer-reported performance specifications and a manufacturer-issued Declaration of Conformity listing EN 61526:2013, the authors did not independently validate the individual devices used in this study against a calibrated reference dosimeter under diagnostic X-ray beam qualities representative of clinical fluoroscopy conditions. Furthermore, the documentation available to the authors did not include individual calibration certificates for the specific devices used, a complete metrological traceability chain, or a comprehensive measurement-uncertainty budget under the specific clinical beam conditions. Consequently, the reported over-apron Hp(10) measurements should be interpreted in the context of these metrological limitations.

## 5 Conclusion

This single-center descriptive and exploratory study characterized occupational radiation exposure among anesthesiology personnel participating in fluoroscopy-guided interventions using real-time dosimetry. Occupational exposure patterns varied across the investigated clinical settings, with mean over-apron Hp(10) ranging from 0.010 to 0.025 mSv per procedure. These differences should be interpreted descriptively within the present dataset and not as evidence of an independent effect of procedural category.

Real-time electronic dosimetry may complement conventional occupational monitoring by providing procedure-level information on exposure patterns, informing radiation-protection practices, and supporting future efforts to reduce occupational radiation exposure.

## Data Availability

The raw data supporting the conclusions of this article will be made available by the authors, without undue reservation.

## References

[1] IARC Working Group on the Evaluation of Carcinogenic Risks to Humans, International Agency for Research on Cancer, World Health Organization, editor. Ionizing Radiation, Part 2: Some Internally Deposited Radionuclides. Lyon, France: IARC Press (2001).

[2] Ainsbury EA Barnard S Bright S Dalke C Jarrin M Kunze S . Ionizing radiation induced cataracts: recent biological and mechanistic developments and perspectives for future research. Mutation Res/Rev Mutation Res. (2016) 770:238–61. doi: 10.1016/j.mrrev.2016.07.010, 27919334

[3] Wang RR Kumar AH Tanaka P Macario A. Occupational radiation exposure of anesthesia providers: a summary of key learning points and resident-led radiation safety projects. Semin Cardiothorac Vasc Anesth. (2017) 21:165–71. doi: 10.1177/1089253217692110, 28190371

[4] Miller DL Vañó E Bartal G Balter S Dixon R Padovani R . Occupational radiation protection in interventional radiology: a joint guideline of the cardiovascular and interventional radiology Society of Europe and the Society of Interventional Radiology. J Vasc Interv Radiol. (2010) 21:607–15. doi: 10.1016/j.jvir.2010.01.007, 20430294

[5] Mendonça RPD Estrela C Bueno MR Carvalho TCASG Estrela LRDA Chilvarquer I. Principles of radiological protection and application of ALARA, ALADA, and ALADAIP: a critical review. Braz Oral Res. (2025) 39:e14. doi: 10.1590/1807-3107bor-2025.vol39.014, 39936713 PMC11808696

[6] Kim KP Miller DL De Gonzalez AB. Occupational radiation doses to operators performing fluoroscopically-guided procedures. Health Phys. (2012) 103:80–99. doi: 10.1097/HP.0b013e31824dae76, 22647920 PMC3951010

[7] Baudin C Vacquier B Thin G Chenene L Guersen J Partarrieu I . Occupational exposure to ionizing radiation in medical staff: trends during the 2009–2019 period in a multicentric study. Eur Radiol. (2023) 33:5675–84. doi: 10.1007/s00330-023-09541-z, 36930262 PMC10326158

[8] Yoder RC Dauer LT Balter S Boice JD Jr Grogan HA Mumma MT . Dosimetry for the study of medical radiation workers with a focus on the mean absorbed dose to the lung, brain and other organs. Int J Radiat Biol. (2022) 98:619–30. doi: 10.1080/09553002.2018.1549756, 30451566 PMC9465996

[9] HERADO Radiation Management Platform Technical Documentation. Athens, Greece: HERADO Technologies (2024).

[10] Olin K Klinga C Ekstedt M Pukk-Härenstam K. Exploring everyday work as a dynamic non-event and adaptations to manage safety in intraoperative anaesthesia care: an interview study. BMC Health Serv Res. (2023) 23:651. doi: 10.1186/s12913-023-09674-3, 37331961 PMC10278258

[11] Khaled MFI Rahman MW Mahabub SMEE Ahmed S Munna MMH Islam MM . Quantifying radiation exposure across cardiac catheterization procedures. Diagnostics. (2026) 16:1636. doi: 10.3390/diagnostics16111636, 42279505 PMC13257294

[12] Fujisawa M Sota M Haga Y Tanaka S Kataoka N Kato T . Real-time monitoring of occupational radiation exposure in nuclear medicine technologists: an initial study. Appl Sci. (2025) 15:11008. doi: 10.3390/app152011008

[13] Saukko E Grönroos JM Salminen P Henner A Nieminen MT. Patient radiation dose and fluoroscopy time during ERCP: a single-center, retrospective study of influencing factors. Scand J Gastroenterol. (2018) 53:495–504. doi: 10.1080/00365521.2018.1445774, 29489436

[14] Goel A Campos A Feger J Goel A. Kilovoltage Peak. Melbourne, Australia: Radiopaedia.org (2014).

[15] Sperry BW Vamenta MS Gunta SP Thompson RC Einstein AJ Castillo M . Influence of body mass index on radiation exposure across imaging modalities in the evaluation of chest pain. JAHA. (2024) 13:e033566. doi: 10.1161/JAHA.123.033566, 38591342 PMC11262536

[16] Narain AS Hijji FY Yom KH Kudaravalli KT Haws BE Singh K. Radiation exposure and reduction in the operating room: perspectives and future directions in spine surgery. WJO. (2017) 8:524–30. doi: 10.5312/wjo.v8.i7.524, 28808622 PMC5534400

[17] Okraku-Yirenkyi NK Bonthu SSR Bhakta H Duyile OO Bernas M. Occupational radiation risk stratification and protection in fluoroscopy-guided surgeons and Interventionalists: a multispecialty structured narrative review. JPM. (2026) 16:50. doi: 10.3390/jpm16010050, 41590542 PMC12842780

[18] Ayoğlu H Ayoğlu FN. Occupational risks for Anaesthesiologists and precautions. Turk J Anaesthesiol Reanim. (2021) 49:93–9. doi: 10.5152/TJAR.2020.219, 33997836 PMC8098723

[19] Maghsoudi B Mortazavi SMJ Khademi S Vatankhah P. Evaluation of radiation exposure pattern and radiation absorbed dose resulting from occupational exposure of anesthesiologists to ionizing radiation. J Biomed Phys Eng. (2017) 7:271–8. doi: 10.1093/aje/kwn171, 29082218 PMC5654133

[20] Clιro E Vaillant L Zhang W Hamada N. ICRP. Radiation detriment calculation methodology. Ann ICRP. ICRP Publication 152. (2022) 51:9–103. doi: 10.1177/01466453221080101 35417894

[21] Krompaß K Mutschler M Grunz JP Thurner A Bley TA Voelker W . Real-time dosimetry in interventional radiology – comparing the occupational radiation exposure in fluoroscopy-guided lower extremity and abdominal procedures. Eur Radiol. (2025) 35:6575–82. doi: 10.1007/s00330-025-11566-5, 40221939 PMC12417226

[22] Sanchez RM Vaño Carruana E Fernández Soto JM. Recording interventionalists’ occupational dose by clinical procedure: a novel approach to radiation safety. CVIR Endovasc. (2026) 9:24. doi: 10.1186/s42155-026-00658-y, 41762425 PMC12950117

[23] Gilvin P Caresana M Bottollier-Depois JF Evaluation of the Impact of the New ICRU Operational Quantities and Recommendations for their Practical Application. Bristol, United Kingdom (2022).

[24] Vanzant D Mukhdomi J. "Safety of fluoroscopy in patient, operator, and technician". In: StatPearls Treasure Island, Florida, USA: StatPearls Publishing (2026). 34033329

[25] Shbeer A. Assessment of the occupational radiation exposure of anesthesia staff in interventional cardiology. RMHP. (2024) 17:1093–100. doi: 10.2147/RMHP.S460054, 38707521 PMC11068050

[26] Helton E Godbe K Bird C Brubacher J. Intraoperative fluoroscopy exposure: a systematic, multi-specialty review of occupational radiation exposure to surgeons. Cureus. (2025) 17:e96001. doi: 10.7759/cureus.96001, 41346880 PMC12673912

[27] Sanchez RM Vano E Fidalgo JR Fernández JM. Percutaneous structural cardiology: are anaesthesiologists properly protected from ionizing radiation? J Radiol Prot. (2020) 1420–1428. doi: 10.1088/1361-6498/abc4d7, 33105107

[28] Drews T Pasic M Juran R Unbehaun A Dreysse S Kukucka M . Safety considerations during transapical aortic valve implantation. Interact Cardiovasc Thorac Surg. (2014) 18:574–9. doi: 10.1093/icvts/ivt560, 24525856

[29] Kara B Şahmaran T. Evaluating the impact of toolbox training on health professionals in radiation environments: a randomized controlled trial. Health Phys. (2026) 130:342–54. doi: 10.1097/HP.0000000000002101, 41631967

[30] Schuster AL Forman HP Strassle PD Meyer LT Connelly SV Lee CI. Awareness of radiation risks from CT scans among patients and providers and obstacles for informed decision-making. Emerg Radiol. (2018) 25:41–9. doi: 10.1007/s10140-017-1557-8, 28948391

[31] Harrison JD Balonov M Bochud F Martin C Menzel HG Ortiz-Lopez P . ICRP publication 147: use of dose quantities in radiological protection. Ann ICRP. (2021) 50:9–82. doi: 10.1177/0146645320911864, 33653178

[32] Damilakis J. Artificial intelligence-powered personalized patient dosimetry in CT. BJR Artif Intell. (2025) 2:ubaf007. doi: 10.1093/bjrai/ubaf007, 42064003 PMC13045712

[33] Little MP Wakeford R Tawn EJ Bouffler SD Berrington De Gonzalez A. Risks associated with low doses and low dose rates of ionizing radiation: why linearity may be (almost) the best we can do. Radiology. (2009) 251:6–12. doi: 10.1148/radiol.2511081686, 19332841 PMC2663578

[34] Stahl CM Meisinger QC Andre MP Kinney TB Newton IG. Radiation risk to the fluoroscopy operator and staff. Am J Roentgenol. (2016) 207:737–44. doi: 10.2214/AJR.16.16555, 28829623

[35] López PO Dauer LT Loose R Martin CJ Miller DL Vañó E . ICRP publication 139: occupational radiological protection in interventional procedures. Ann ICRP. (2018) 47:1–118. doi: 10.1177/0146645317750356, 29532669

[36] Valentin J. ICRP publication 103 the 2007 recommendations of the international commission on radiological protection. Ann ICRP. (2007) 37:9–34. doi: 10.1016/j.icrp.2007.10.003, 18082557

[37] Menzel HG Harrison J. Dosimetric quantities in radiological protection and risk assessment. J Radiol Prot. (2012) 32:N41–6. doi: 10.1088/0952-4746/32/1/N41, 22395228

[38] Halmes NC. Reevaluating Cancer risk estimates for short-term exposure scenarios. Toxicol Sci. (2000) 58:32–42. doi: 10.1093/toxsci/58.1.32, 11053538

